# Feasibility study to evaluate the MMI Symani robotic system for microsurgical techniques in an in-vitro circulation model

**DOI:** 10.1016/j.jvscit.2025.101767

**Published:** 2025-03-05

**Authors:** Melanie Rusch, Grischa Hoffmann, Rouven Berndt, René Rusch

**Affiliations:** a Clinic of Vascular and Endovascular Surgery, University Hospital Schleswig-Holstein, Kiel, Germany; b Kurt-Semm-Center for Laparoscopic and Robotic-Assisted Surgery, University Hospital Schleswig-Holstein, Kiel, Germany

**Keywords:** Minimally invasive surgery, Microsurgery, Robotic surgery, Cardiovascular surgery, Bypass surgery

## Abstract

The Symani Surgical System is a novel robotic microsurgical platform. This study investigated its feasibility for arterial reconstructive techniques in an in vitro model. Two senior surgeons without preliminary expertise in robotic surgery performed different techniques in a porcine artery circulation model (bypass, patch plastic, and direct closure of arteriotomy). There was an overall improvement in procedure times with a minor leakage rate and upper range values in the qualitative assessment of anastomoses. In this model, the Symani Surgical System seems to be suitable for the robotic-assisted conductance of microsurgical procedures on arterial vessels.

Robot-assisted surgery has become an important part of surgery in many fields owing to the minimally invasive access, improved visualization, and precise movements.^1^^,^^2^ The Symani Surgical System (MMI, Medical Microinstruments, Jacksonville, FL) is a novel microsurgical robotic platform, used in lymphatic surgery and microvascular anastomoses in the field of plastic and maxillofacial surgery, but also recently in cardiovascular surgery.^3^, ^4^, ^5^

The use of the MMI system might provide additional opportunities for surgery in small vascular calibres as in the femoropopliteal segment owing to its three-dimensional (3D) visualization and the precise downscaling of movements.^6^^,^^7^ In this study the feasibility of the MMI system for microsurgery on arteries, in an vitro experimental setting was investigated.

## Methods

**Fig 1:**
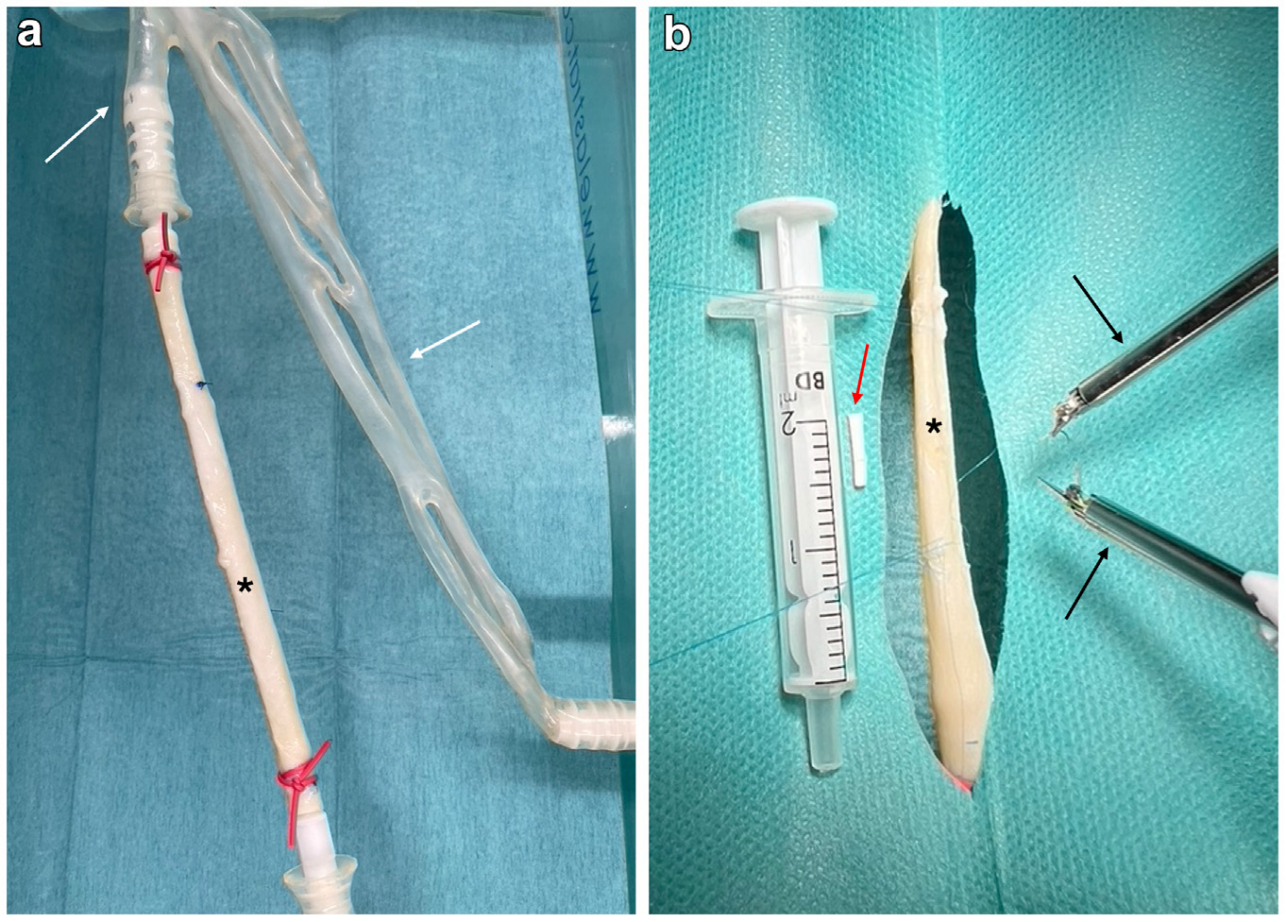
Experimental setup of the Symani Surgical System in the circulation model. **(A)** Positioning of the porcine artery (∗) in the replaceable vascular-phantom tree (white arrows). **(B)** Positioning of the robotic arms (black arrows) in the distance to the surgical setup (∗) and the artificial bypass (red arrow).

**Fig 2:**
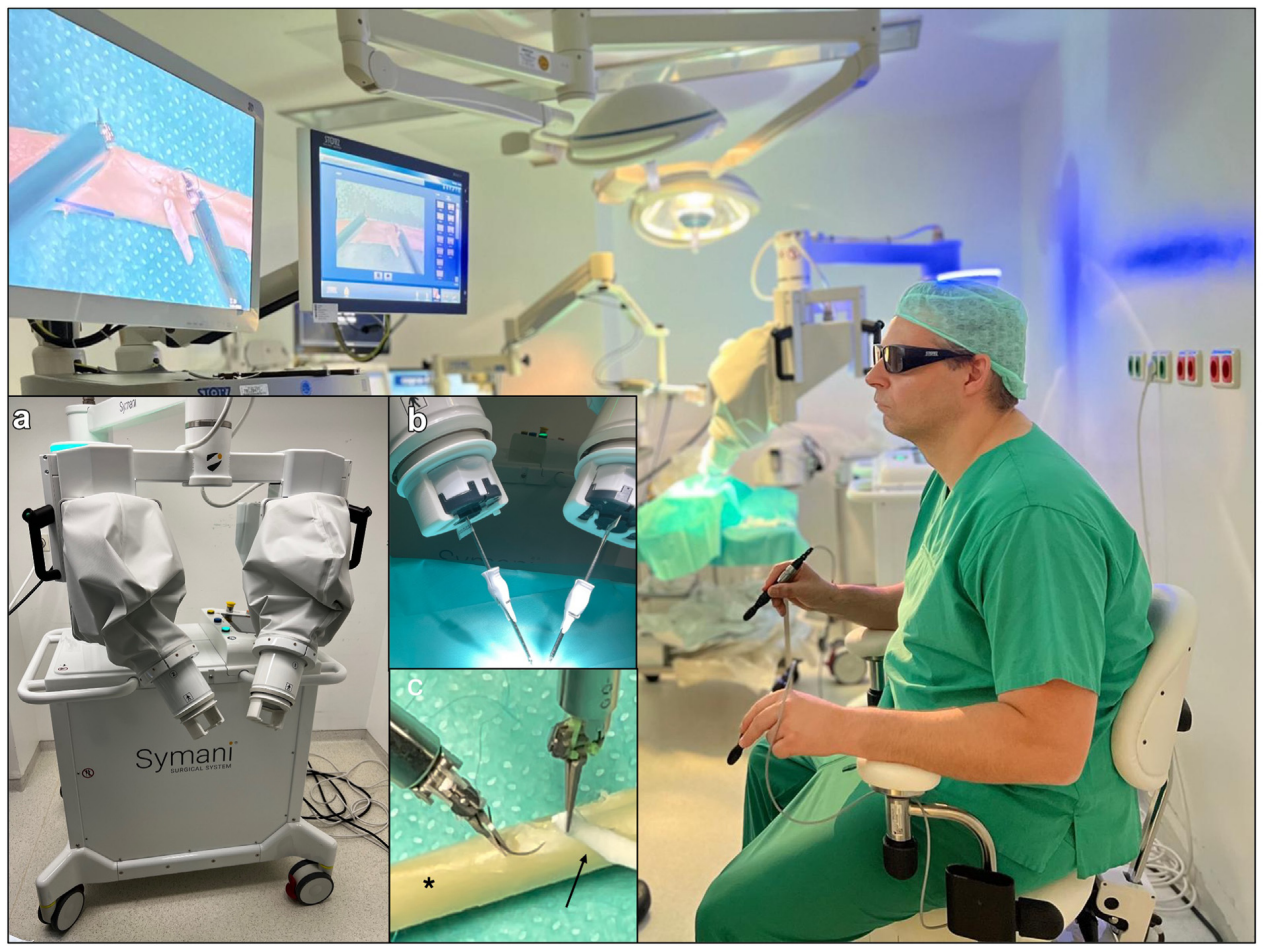
Experimental setup. Remote control of the robotic platform **(A)** with exoscope using three-dimensional (3D) vision. **(B)** Micromanipulators holding the NanoWrist instruments. **(C)** Bypass anastomosis (black arrows) on the porcine artery (∗).

The study was approved by the local ethics committee of the University Medical Center Schleswig-Holstein, Kiel, Germany (lD: D 642/23). A mechanical volume-controlled pulsatile pump with an average flow rate of 2.5 L/min was connected to segments of porcine iliac arteries fixed in an acrylic resin-based model (ELASTRAT, Geneva, Switzerland). The working fluid consisted of a crystalloid solution (60% glucose solution).^8^ The Symani Surgical System with the micromanipulators holding the NanoWrist instruments was placed in front of the circulation model (Fig 1). Surgery was performed in a remote manner (Fig 2) in combination with a 3D exoskope (VITOM 3D system, Karl Storz, Tuttlingen, Germany). The anastomosis times of each surgeon were documented and the surgical performance evaluated. After declamping the anastomoses were inspected for leakage visually.

**Fig 3:**
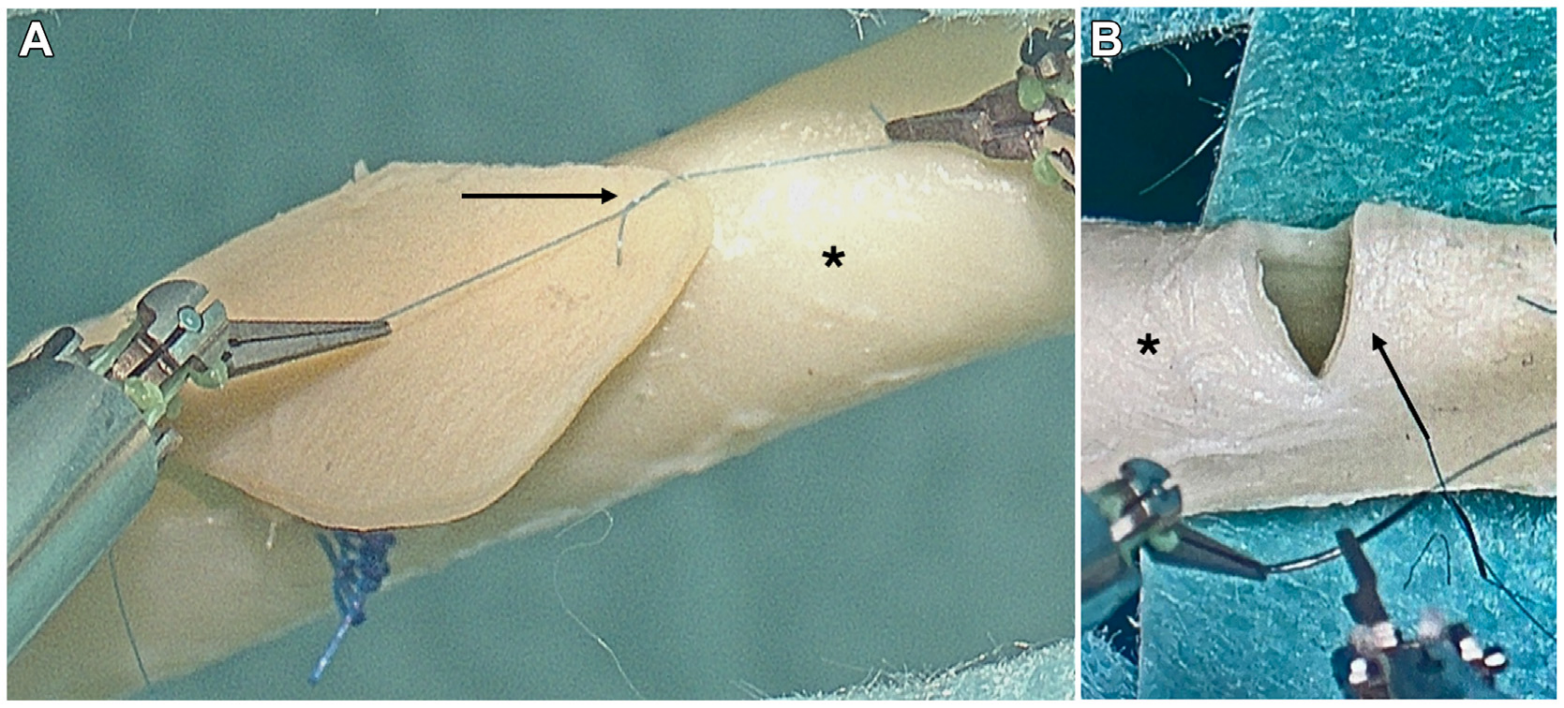
**(A)** Patch reconstruction (black arrow) on the porcine artery (∗) using the single-button suture technique. **(B)** Illustration of the transverse incision (black arrow) in preparation for direct closure.

The reconstructions were performed by two senior cardiovascular surgeons without preliminary experience in robotic surgery who underwent a baseline training of 12 hours to acquire the mandatory certification for the robotic system. A total of 18 reconstructions on the clamped arteries were performed: 3 patch reconstructions, 3 direct closures of a transverse incision, and 3 bypass anastomoses. For patch reconstruction and bypass anastomosis, a 15-mm longitudinal incision was created (Fig 2, *C*). Patch reconstruction was performed with bovine pericardium using a single suture technique. For bypass creation a 1 mm artificial graft (WetLab Inc., Otsu-city Shiga, Japan) was anastomosed with running sutures (Fig 3, *A*). A transverse incision of 10 mm was created for direct closure with three single sutures (Fig 3, *B*). All reconstructions were performed with 9/0 Prolene sutures (Ethicon, Johnson & Johnson Medical GmbH, Norderstedt, Germany). After completing the reconstruction and declamping the flow was restarted.

All statistical analysis were performed with the PRISM 8.4.3 software (GraphPad, San Diego, CA). Values of continuous data are presented as mean ± standard deviation. Evaluation of the completed anastomosis was assessed by applying the slightly modified Northwestern Objective Microanastomosis Assessment Tool, focusing on sufficiency and quality of reconstruction (items XII-XIV; range, 0-15) and in vivo viability grading (pass/fail) made by two independent surgical trained observers via video assessment.^9^

## Results

Vascular repair was performed without any technical failures. The placement of the micromanipulators, such as instruments, and 3D visualization was easily feasible and reproduceable. With the different reconstruction techniques, both surgeons showed slight improvement of anastomosis times during the series. The time for bypass anastomosis for surgeon 1 decreased from 12.5 to 10.5 minutes (15.3%) and from 13.3 to 10.4 minutes (21.8%) for surgeon 2. For patch reconstruction, times decreased from 12.3 to 8.2 minutes (33.3%) and from 12.5 to 9.4 minutes (24.8%), respectively. The time for incision closure decreased for surgeon 1 from 6.3 to 4.2 minutes (33.3%) and for surgeon 2 from 6.5 to 5.2 minutes (20,0%). The leakage test showed improved quality with increasing surgical experience. Direct closures and patch reconstructions showed no major leakage or anastomosis failure, although minor leaks were detected in two bypass anastomoses (Table I). The evaluation of the Symani Surgical System showed considerable advantages in the assessment of the stitch distances, particularly in the 3D visualization. Depending on the diameter of the incision, the stitch spacing could be reduced to a maximum of six stitches for both bypass anastomosis and patch reconstruction for passing the leakage test successfully. Qualititative assessment using the Northwestern Objective Microanastomosis Assessment Tool for surgical handling and sufficiency showed values in the upper range with good user feedback (Table II).

**Table I:** Leakage test after completion of the anastomosis for each attempt

	Leakage test		
	Attempt 1	Attempt 2	Attempt 3
Bypass reconstruction			
Surgeon 1	pos	neg	pos
Surgeon 2	neg	pos	pos
Patch reconstruction			
Surgeon 1	pos	pos	pos
Surgeon 2	pos	pos	pos
Direct closure			
Surgeon 1	pos	pos	pos
Surgeon 2	pos	pos	pos

*neg,* Anastomosis leaking; *pos,* anastomosis tight.

**Table II:** Northwestern Objective Microanastomosis Assessment Tool (*NOMAT*) for surgical handling and sufficiency of the vascular anastomosis

	MMI surgery
Mean NOMAT (modified)	10.5
In vivo viability	16 pass, 2 fail
Mean anastomosis time, minutes	10:59 ± 01:34

## Discussion

In contrast with other surgical fields, robotic-assisted surgery has not been established in vascular surgery.^10^^,^^11^ The Symani Surgical System is the first robotic platform especially designed for microanastomoses.^12^ Most recently, an experimental study on porcine coronary vessels demonstrated the feasibility of an in vitro model of coronary artery surgery.^3^ In vascular surgery, potential areas of application could include small-caliber reconstructions and anastomoses of the small peripheral vessels. The improved 3D visualization and the high-precision movement options with adapted speed profiles represent a significant technical advantage in vascular reconstruction under difficult access conditions.^13^

This study describes the application of the MMI system on different reconstruction techniques on arteries of medium to smaller caliber. The application of the system in a challenging simulated anatomical region was uncomplicated and the two robotic arms could be used in a very direct and targeted way. The optimized 3D visualization and precise direction of movements allowed a better spatial perception of the surgical field.

The Symani Surgical System proved to be potentially feasible for small diameter anastomoses in the peripheral vascular region.^3^ Owing to the limited access routes, for example, in the crural segment, 3D visualization with the possibility of multiple scale magnification represents a clear technical advantage.^13^^,^^14^ Various studies have described the importance of optimal visualization during robot-assisted procedures and the possible effect on operating time and complications.^15^^,^^16^ In our study, the users were able to perform very direct and targeted movements when creating the anastomoses in the circulation model. The improved spatial perception enabled highly precise placement of stitches for the various reconstructions. The MMI system generated a good optical tissue feedback—in particular, intima and media damage could be detected and prevented. This optimized visual feedback of the tissue can sensitize to more precise movement sequences, which enable successful reconstructions as the learning curve advances.^17^^,^^18^

The Symani Surgical System continues to be a very intuitive and highly suitable instrument for creating microanastomoses. Based on its previous application in free flap reconstruction using the posterior tibial, anterior tibial, and radial arteries as recipient vessels, we aim to use the Symani Surgical System in the near future in patients for procedures on the crural and pedal vessels or in the creation of dialysis accesses.^4^ Nevertheless, the acquisition and application costs for the use of this robotic platform are significantly higher than for conventional vascular surgery, with an unclear reimbursement situation. In addition, randomized studies are needed to further evaluate the use of the system, particularly in the cardiac and vascular fields.^19^^,^^20^ However, 3D visualization offers great potential for selected areas of application in restricted minimally invasive surgical fields owing to the improved perspective and more precise tissue imaging.

## Conclusions

Robot-assisted techniques have not been established as a standard procedure in vascular surgery owing to a lack of application fields. Vascular procedures on very small vessels in regions with difficult access will potentially benefit from improved 3D visualization, microsurgery, and the downscaling of movements using the robot-assisted system. Peripheral reconstructive vascular surgery could be a potential area of application for the Symani Surgical System in the future. To define further areas of application controlled, randomized studies are mandatory.

## Funding

None.

## Disclosures

The Symani Surgical System was provided to the members of the Kurt-Semm-Center for Laparoscopic and Robotic-Assisted Surgery in Kiel for clinical and scientific use with no competing interests and no funding was provided.
